# Multi-gigahertz, femtosecond Airy beam optical parametric oscillator pumped at 78 MHz

**DOI:** 10.1038/srep43913

**Published:** 2017-03-06

**Authors:** A. Aadhi, Varun Sharma, N. Apurv Chaitanya, G. K. Samanta

**Affiliations:** 1 Photonic Sciences Lab., Physical Research Laboratory, Navarangpura, Ahmedabad 380009, Gujarat, India

## Abstract

We report a high power ultrafast Airy beam source producing femtosecond pulses at multi-gigahertz (GHz) repetition rate (RR). Based on intra-cavity cubic phase modulation of an optical parametric oscillator (OPO) designed in high harmonic cavity configuration synchronous to a femtosecond Yb-fiber laser operating at 78 MHz, we have produced ultrafast 2D Airy beam at multi-GHz repetition rate through the fractional increment in the cavity length. While small (<1 mm) crystals are used in femtosecond OPOs to take the advantage of broad phase-matching bandwidth, here, we have exploited the extended phase-matching bandwidth of a 50-mm long Magnesium-oxide doped periodically poled LiNbO_3_ (MgO:PPLN) crystal for efficient generation of ultrafast Airy beam and broadband mid-IR radiation. Pumping the MgO:PPLN crystal of grating period, Λ = 30 μm and crystal temperature, T = 100 °C using a 5-W femtosecond laser centred at 1064 nm, we have produced Airy beam radiation of 684 mW in ~639 fs (transform limited) pulses at 1525 nm at a RR of ~2.5 GHz. Additionally, the source produces broadband idler radiation with maximum power of 510 mW and 94 nm bandwidth at 3548 nm in Gaussian beam profile. Using an indirect method (change in cavity length) we estimate maximum RR of the Airy beam source to be ~100 GHz.

Airy beam, since its first demonstration^1^ in 2007, has created profound impact in variety of fields in science and technology. In particular, due to its peculiar properties such as self-acceleration (ballistic propagation dynamics), non-diffraction (invariant intensity profile along propagation) and self-healing (restoration of its canonical form even after obstruction by small objects), Airy beams find many applications including optically mediated particle clearing, curved plasma wave-guiding, long distance communication, micro-particle manipulation, nonlinear frequency conversion and high resolution microscopy^2^,^3^,^4^,^5^. Typically, the Airy beams are generated through cubic phase modulation and subsequent Fourier transformation of a laser beam in Gaussian spatial distribution^1^. However, the Airy beams generated through different techniques suffer from common drawbacks of limited or no wavelength tunability and low acceleration lengths, power and energy^6^,^7^. Given that the optical parametric oscillators (OPOs)^8^ especially in singly resonant (SRO) configuration produces widely tunable, high power/energy optical radiation, the recent demonstration of Airy beam OPOs in both continuous-wave (cw)^9^ and picosecond^10^ time scales have produced high power/energy optical radiation in 2-D Airy intensity distribution existing over a long propagation distance (>2 m) and also tunable across wide wavelength range with controlled beam acceleration. However, some of the applications such as curved plasma channel, supercontinuum generation and filamentation^3^,^11^,^12^ require Airy beams in femtosecond timescales along with high average powers. Furthermore, future applications in high speed telecommunication systems^13^, photonic switching^14^, and time-resolved spectroscopy^15^ require femtosecond Airy beams at high repetition rate (RR). To address such requirement one can in principle generate Airy beam from synchronously pumped femtosecond OPOs. While multi-GHz OPO is possible in picosecond time scale by using fractional cavity configuration^16^, where the resonating pulse makes large number of oscillations inside the cavity before meeting the pump pulse and amplification, the increase in RR requires high power pump lasers and/or large parametric gain to overcome the cavity losses of the resonating signal radiation. Due to unavailability of femtosecond laser with high average power (>5 W) and use of thin nonlinear crystal to accommodate the pump spectra in parametric gain bandwidth for efficient nonlinear conversion, the RR of the femtosecond OPOs has been restricted to few GHz^17^. Here we report, for the first time to the best of our knowledge, a multi-GHz optical parametric oscillator producing femtosecond pulses in 2D Airy intensity distribution. Using the extended phase-matching^18^ characteristics of 50-mm-long MgO-doped periodically poled LiNbO_3_ (MgO:PPLN) crystal pumped by an ultrafast fiber laser at 1064 nm in a novel high harmonic fractional SRO cavity configuration, we have produced femto-second pulses at a RR of 100 GHz with average power of ~684 mW in 2D Airy intensity profile at 1525 nm. Additionally, the source provides broadband output radiation with power of 510 mW and bandwidth of 94 nm centred at 3548 nm in the Gaussian beam profile. The generic harmonic fractional cavity configuration can be used for any ultrafast lasers to generate high RR optical radiation at different wavelengths across the electromagnetic spectrum.

## Experimental setup

### Airy beam generation

**Figure 1:**
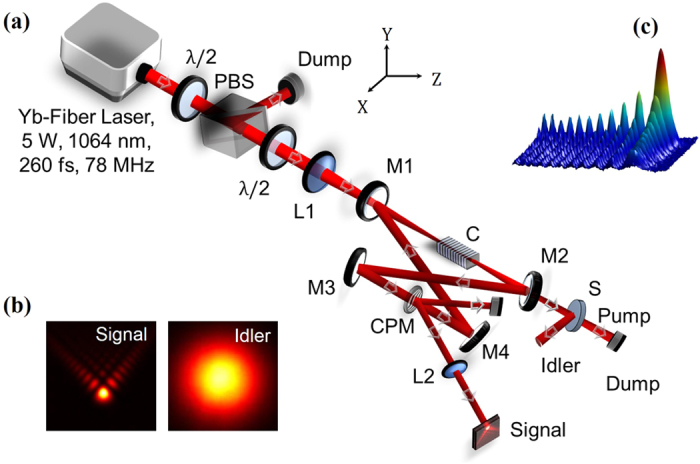
Multi-GHz repetition rate, femto-second Airy beam optical parametric oscillator. (**a**) Schematic of the experimental setup. M1-4; mirrors, L1-2; lens, λ/2; half-wave plate, PBS; polarization beam splitter cube, C; MgO:PPLN crystal for OPO, CPM; cubic phase mask, S; wavelength separator. (**b**) Intensity profile of the Airy beam signal and Gaussian beam idler radiation. (**c**) 3-D illustration of the generated Airy beam.

The schematic of the experimental setup for the high RR Airy beam source is shown in Fig. 1. An ultrafast Yb-fiber laser (Fianium, FP1060-5-fs) of 5 W average power and spectral width of ~15 nm centred at 1064 nm is used as a pump source to SRO. The pump laser provides ultrafast pulses of width 260 fs at a RR rate of 78 MHz. The fundamental laser has a TEM_00_ spatial-beam profile with *M*^*2*^ < 1.3. Operating the fiber laser at its highest power to access its optimum performance in terms of temporal width, a combination of half wave plate (λ/2) and a polarization beam splitter cube (PBS) are used to control the laser power to the nonlinear crystal. The SRO is configured in a compact four mirror ring cavity with two plano-concave mirrors, M1-M2, with radius of curvature, *r* = 100 mm and two plane mirrors, M3-M4. All the cavity mirrors have high transmission (T > 90%) for pump wavelength at 1064 nm, and idler wavelengths across 2100–4000 nm and high reflectivity (R > 99%) for the signal wavelength across 1400–2000 nm. The physical distance between the mirrors, M1 and M2 is maintained constant at 138 mm throughout the experiment. A 50 mm long and 8 × 1 mm^2^ aperture multi-grating MgO-doped periodically poled LiNbO_3_ (MgO:PPLN) (HC Photonics, Taiwan) crystal with grating period, Λ_c_ varying from 28.5 μm to 32 μm in 0.5 μm increment, is used as nonlinear crystal for the OPO. A lens L1 of focal length *f* = 100 mm is used to focus the pump beam to an estimated beam waist radius of *w*_*p*_ = 43 μm corresponding to a confocal parameter of *b* = 24 mm. From the ABCD ray matrix formalism we designed the cavity length of 3846 mm (for *Q* = 1) resulting signal and idler beam waist radius *w*_*s*_ = 52 μm and *w*_*i*_ = 79 μm respectively. A cubic phase mask (CPM) in the form of a binary grating with amplitude transmittance given by refs 9 and 10 is used for cubic phase modulation of the Gaussian beam as required for Airy beam generation. The CPM fabricated at the centre of a 3-mm-thick fused silica substrate of diameter 25.4 mm over a region of 2 × 2 mm^2^ aperture placed between mirror, M3 and M4 is used for phase modulation of the resonant signal beam. The grating has N = 100 lines in L = 2 mm resulting a carrier period of 20 μm. The cubic phase modulation strength of CPM is estimated to be, c_o_ = 5.77/mm^9^. The phase mask has 0^th^ order diffraction efficiency of ~96% at ~1523 nm corresponding to ~4% output coupling of the intra-cavity signal in two first order diffracted beams in the form of cubic phase modulated Gaussian beams. A lens (L2) of focal length, *f* = 300 mm Fourier transforms the 1^st^ order cubic phase modulated Gaussian beam into Airy beam. Like our previous report^9^, here, the cubic phase mask CPM is rotated by 45° counter clockwise with respect to its conventional orientation^10^ to produce Airy beam acceleration in the direction perpendicular (Y-direction) to the optical table. Such an arrangement allows for the study of Airy beam acceleration over a long propagation length (~2 m) without use of any reference beam.

### Broadband mid-IR generation

In parametric process, the spectral width of the pump (acceptance bandwidth (BW)) contributing to efficient generation of signal and idler, is restricted by the group-velocity-mismatch (GVM) between the signal and idler radiation. In such case the pump acceptance BW is inversely proportional to the crystal length. Therefore, widely established techniques^19^,^20^ for ultrafast broadband generation relies on parametric down conversion of ultrafast laser in thin second order nonlinear crystals. While a large spectral acceptance bandwidth (BW) of thin nonlinear crystals allows conversion of broad BW of the pump laser into a broad spectral range^21^, the dependence of crystal length on parametric gain reduces overall conversion efficiency resulting in a trade-off between the BW of the generated radiation and the length of the nonlinear crystal. However, it is evident from the expansion of the phase-mismatch of the interacting radiation in parametric generation as explained in ref. 18, that under perfect phase-matching, in absence of GVM among the interacting waves, the pump acceptance BW is inversely proportional to the square-root of the crystal length. While group velocity matching is achieved at the degeneracy point, where signal and idler share the same wavelength and polarization; interestingly, however, for some of the nonlinear crystals, there are other group velocity matching wavelengths. In MgO:PPLN, one can also identify such a condition to happened for pump beam at ~1059 nm and signal and idler wavelengths around 1525 nm and ~3476 nm respectively at grating period, Λ_c_ = 30 μm and crystal temperature, T = 100 °C^18^. A 50-mm-long crystal has pump acceptance BW calculated to be ~103.9 cm^−1^ at a pump wavelength centred at 1059 nm^18^. The ultrafast Yb-fiber laser of the current experiment has a spectral width of ~133.49 cm^−1^ (~15 nm) centred at 1064 nm. The pump acceptance bandwidth as calculated in ref. 18 for a centre wavelength of 1059 nm is still within the 15 nm bandwidth of the pump laser. Therefore, we have used Λ_c_ = 30 μm grating of the 50-mm-long MgO:PPLN crystal at an operating temperature of T = 100 °C and transferred the pump BW for efficient generation of ultrafast broadband output radiation in the mid-IR wavelength range.

### Multi-GHz repetition rate operation

Due to unavailability of suitable lasers to produce output pulses at high rep. rate from an OPO through synchronous pumping^22^, the high rep. rate OPOs are realized through two common experimental schemes: high harmonic^23^ and fractional incremental^16^ cavity configurations. In case of high harmonic cavity, where the OPO cavity length is 1/*N* times that of the pump laser to produce an output pulse train with *N* times the RR of the laser^23^. However, shortening of the OPO cavity length limits the practical realization of such a scheme to low RR operation. On the other hand, in case of fractional OPO cavity configurations, the OPO cavity length is *n/N (n* is an integer, *n* > *N*, and with no common divisor^17^) times the cavity length of the pump laser to produce output pulse train at N times the pump RR. While increase in cavity length in such cavity configuration allows incorporation of additional optical components as required for different OPOs, such cavities are susceptible to external perturbations such as mechanical instability, air turbulence and temperature fluctuations limiting the overall performance of the high rep. rate OPOs. To overcome such problems, here, we present a new experimental scheme to generate pulse trains in the multi-GHz RR by incorporating the advantages of both techniques. In this new method, to generate the *N*^ th^ harmonic of the pump RR, we set the OPO cavity length to be (1*/Q*) times the pump laser cavity length, *L*_*p*_, to produce the *Q*^th^ harmonic cavity and increase the cavity length by ΔL = *L*_*p*_*/(PQ*). Here, *P* and *Q* are positive integers, and *N* = *PQ*. For this new OPO cavity length, *L*_*opo*_ = *L*_*p*_*/Q* + ΔL = *L*_*p*_ (*P* + 1)*/PQ*, if (*P* + 1)*/Q* is not an integer, the intracavity resonant pulse makes *PQ* number of oscillations before meeting the next (*P* + 1)^th^ pump pulse. Since in every round trip the signal pulse sees the output coupler in the cavity, some part of the signal will be emitted from the OPO resulting in output pulses at PQ times the RR of the pump laser. Like the increase of cavity length, as the case of the present scheme, one can also get the similar result by reducing the cavity length by ΔL = *L_p_/(PQ*) from its length synchronous to the *Q*^th^ harmonic of the pump RR. For *n* = (*P* + 1)*/Q* having integer values, the present scheme represents the scheme of ref. 17, however, *n* is smaller than *P*. For *Q* = 1, the experimental scheme represents the scheme of ref. 16. As *PQ* *>* (*P* + 1) for *Q* *>* 1, the OPO cavity length (*L*_*opo*_) in the current experimental scheme is much smaller than the cavity length, *L*_*p*_, synchronized to the RR of the pump to produce output pulses at high repetition rate. Additionally, the flexibility in the selection of *Q* values gives an additional handle to design optimum OPO cavity length to incorporate intra-cavity optical elements while generating high RR pulses.

## Experimental results and Discussion

### Airy beam characteristics

**Figure 2:**
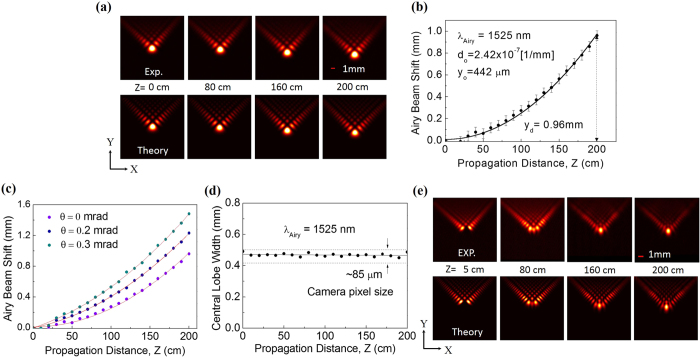
Spatial characteristics of the multi-GHz RR, femto-second Airy beam OPO. (**a**) Experimental (first row) and theoretical (second row) images of 2D intensity distribution of the Airy beam source at wavelength of 1525 nm along the propagation at Z = 0, 80, 160 and 200 cm. (**b**) Airy beam shift along the propagation. (**c**) Airy beam shift along propagation for different launching angle, θ. (**d**) Variation in the width of the central lobe of Airy beam along propagation. (**e**) Experimental (first row) and theoretical (second row) images of 2D intensity profile of Airy beam showing self-healing.

The spatial intensity distribution of the signal beam at wavelength, λ = 1525 nm and at a RR = 2.5 GHz as shown in Fig. 1(b) appears to be the pattern of an Airy beam, however, for further confirmation we have studied acceleration, non-diffraction, and self-healing properties. The results are shown in Fig. 2. To verify acceleration we have recorded 2-D intensity profile of the first order diffracted beam [see Fig. 1(a)] along the propagation direction with *Z* = 0 as the Fourier plane of the lens (*f* = 300 mm) using a large area pyroelectric array camera of pixel size 85 × 85 μm^2^. The first row of Fig. 2(a) shows the experimental images of the transverse profile of the Fourier transformed cubic phase modulated Gaussian beam at propagation distances *Z* = 0, 80, 160, and 200 cm. A close look to the images reveals that the beam has a symmetric intensity distribution with respect to the central lobe and the beam moves in Y-direction along the propagation distance, *Z*. The theoretical images of the beam as shown in the second row of Fig. 2(a), calculated from experimental parameters, show a close agreement with experimental images. However, for quantification of beam acceleration and related parameters, we have recorded the shift in the central lobe position of the beam with propagation from *Z* = 0 with results shown in Fig. 2(b). It is clearly observed that the beam follows a parabolic trajectory (transverse acceleration) along propagation, *Z*, confirming the generation of Airy beam. The Airy beam has a maximum transverse shift of ~0.96 mm over a propagation of 200 cm. For symmetric 2D Airy beam (having symmetric intensity distribution on either side of the central lobe) of wavelength, λ, the transverse acceleration along y-axis can be expressed as^24^


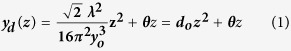


where, *d*_*o*_ and *y*_*o*_ are the deflection coefficient and characteristic length respectively and *θ* is the launching angle of the Airy beam. Using Eq. (1) to the results of Fig. 2(b) for *θ*=0, we found the Airy beam parameters d_o_ and y_o_ to be 2.41 × 10^−7^[1/mm] and 442 μm respectively. The characteristic length, *y*_*o*_, of the Airy beam can be measured by fitting theoretical expression of 1D Airy beam to 1D line profile of the Airy beam images and measuring the distance between the central and the first maxima^25^. Using such direct measurement technique we found the characteristic length, *y*_*o*_, of the Airy beam to be 434 μm, in close agreement with the one (*y*_*o*_ = 442 μm) calculated from d_o_. As evident from Eq. (1), the transverse acceleration of the Airy beam depends on the launching angle, *θ*. We experimentally observed (see Fig. 2c) that with the change in the launching angle from *θ* = 0 to 0.3 mrad, the maximum transverse shift of the Airy beam is varying from 0.96 to 1.48 mm. However, as expected, the characteristic length, *y*_*o*_, (=442 μm) of the Airy beam is independent of the launch angle, *θ*.

To verify non-divergence property of the Airy beam we have recorded the spatial width (full width at half-maximum, FWHM) of the central lobe of the beam (in 1D) along propagation with the results shown in Fig. 2(d). As evident from Fig. 2(d), the central lobe width of the Airy beam varies from 450 μm to 487 μm over a propagation distance of *Z* = 200 cm and the beam divergence is well below the camera pixel size (~85 μm) proving the non-divergence property of the Airy beam even after a propagation distance of 2 m. We have also studied the self-healing property of the Airy beam by blocking the main lobe using a knife edge at the Fourier plane (*Z* = 0) and recording the beam intensity distribution along the propagation with the results shown in Fig. 2(e). The first row of Fig. 2(e) shows the experimental images recorded at *Z* = 5 cm, 80 cm, 160 cm and 200 cm. As evident, the Airy beam has no central lobe at *Z* = 5 cm, however, during propagation, the beam shows signs of healing in the beam shape with a complete regeneration at a distance ~160 cm and maintains its shape with further propagation. Using the experimental parameters we have numerically simulated images of the intensity distribution for the self-healing properties of Airy beam. The theoretical images of the Airy beam intensity distribution, as shown in second row of Fig. 2(e), simulated using the experimental parameters show a close agreement with the experimental images. The presence of all three peculiar properties (self-acceleration, non-divergence and self-healing) proves successful generation of high quality Airy beam from OPO.

### Temporal and spectral characteristics of the broadband Airy beam OPO

**Figure 3:**
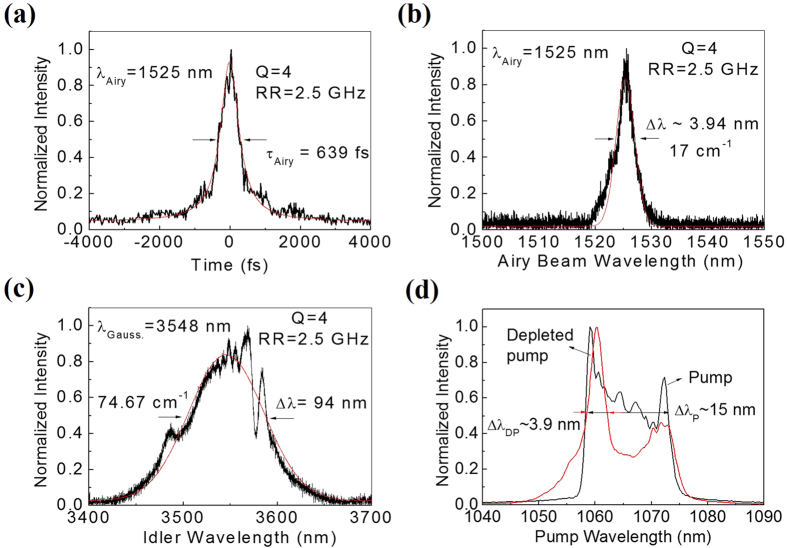
Temporal and spectral characteristics of multi-GHz RR, femto-second Airy beam OPO. (**a**) Temporal width (FWHM) and (**b**) Spectral width of the Airy beam pulses of rep. rate of 2.5 GHz at 1525 nm. (**c**) Broad spectral width of the idler beam in Gaussian spatial profile at 3548 nm. (**d**) Spectral distribution of the pump radiation for OPO off (black line) and OPO on (red line).

After confirming the generation of Airy beam radiation from the OPO at signal wavelength, λ_Airy_ = 1525 nm we operated the OPO by selecting the grating period, Λ_c_ = 30 μm of the 50-mm-long MgO:PPLN crystal at a temperature, T = 100 °C and measured the temporal and spectral characteristics of the Airy beam source with the results shown in Fig. 3. Operating the Airy beam OPO at a RR of 2.5 GHz we measured the temporal width (full-width at half-maximum, FWHM) of the Airy beam radiation using an autocorrelator (Femtochrome, FR-103 XL) to be ~987 fs. Assuming output pulses in sech^2^ pulse shapes and multiplying the autocorrelation width with deconvolution factor of ~0.65, we find that the Airy beam pulse has an average temporal width (FWHM) of τ_Airy_∼639 fs (see Fig. 3a) at a RR of 2.5 GHz. We also measured the spectral width of the Airy beam signal and the Gaussian beam idler radiation using high resolution wavemeter (721, Bristol) with the results shown in Fig. 3(b and c) respectively. The signal [see Fig. 3(b)] and idler [see Fig. 3(c)] radiation have spectral width (FWHM) of Δλ_Airy_~3.94 nm (~17 cm^−1^) centred at λ_Airy_ = 1525 nm and Δλ_Gauss_ ~94 nm (~74.7 cm^−1^) centred λ_Gauss_ = 3548 nm respectively, clearly showing the generation of broadband radiation in the mid-IR wavelength range in Gaussian spatial profile [see the image of idler in Fig. 1(b)]. To understand the broadband generation and further possibility of increasing the spectral width of the mid-IR radiation we have recorded the pump spectra, as shown in Fig. 3(d), for OPO-off [see black line of Fig. 3(d)] and OPO-on [see red line of Fig. 3(d)]. From the black and red lines of Fig. 3(d) representing the spectral distribution of the input and depleted pump respectively, we measured the spectral width (FWHM) of the input pump and the depleted pump to be Δλ_P_~132.5 cm^−1^ (~15 nm) and Δλ_DP_~34.4 cm^−1^ (~3.9 nm) respectively. The spectral acceptance BW of the 50 mm long MgO:PPLN crystal (Λ_c_ = 30 μm, T = 100 ± 0.1 °C) of Δλ_P_ −Δλ_DP_~98 cm^−1^ (~11.1 nm), measured from the undepleted pump bandwidth, is slightly smaller than its theoretical value of ~103.9 cm^−1^ as reported in ref. 18. Such discrepancy can be attributed to the Sellmeier equation^26^ used for the theoretical study. Similarly, the discrepancy between the pump acceptance BW and the spectral BW of output beams as presented in Fig. 3(b and c) can be attributed to the low resolution (~0.5 nm) of the spectrometer (HR 4000, Ocean Optics) used to measure the pump spectra. The time-bandwidth product of the Airy beam pulses in *sech*^*2*^-shape is measured to be ΔτΔν~0.32, close to its transform limit (~0.315). The direct generation of femtosecond pulses from a 50-mm long MgO:PPLN crystal indicates strong interplay between the dispersion properties of interacting waves resulting in a vanishing GVM of the crystal at λ_Airy_ = 1525 nm. An increase of the harmonic number to produce Airy beam pulses at high RR does not show any substantial change in the time-bandwidth product. Due to the absence of a suitable autocorrelator, we did not measure the pulse width of the Gaussian idler beam in the mid-IR wavelength.

### Performance of Airy beam source at high repetition rate

**Figure 4:**
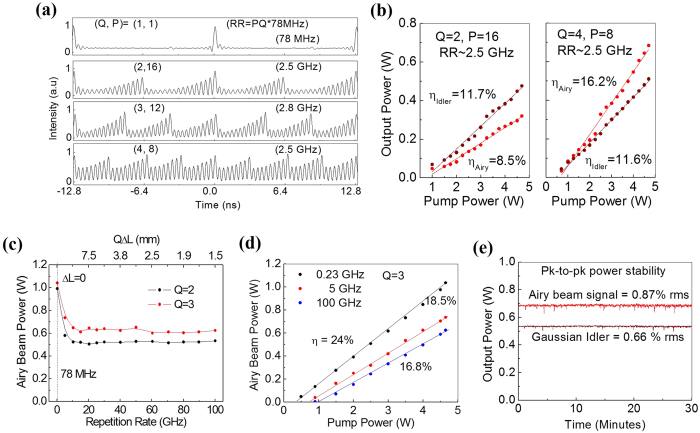
Performance parameters of the Airy beam source at high RR. (**a**) Oscilloscope trace of the Airy beam pulses for different values of *P* and *Q*. (**b**) Variation of output power of the Airy beam OPO of RR = 2.5 GHz for *Q* = 2 and *Q* = 4. (**c**) Dependence of Airy beam power on its RR. The RR of source with *ΔL* = 0 is 156 MHz and 234 MHz for *Q* = 2 and 3 respectively. (**d**) Power scaling of the Airy beam OPO at different RR. (**e**) Stability of the Airy beam source for *Q* = 4 and *P* = 8. The lines are a guide to the eye.

We have also studied the performance of the Airy beam source at high RR with the results shown in Fig. 4. The Fig. 4(a) shows the Airy beam pulse train of rep. rate *N*^ th^ (=*PQ*) harmonic to the pump RR (=78 MHz) recorded using an InGaAs photo-detector (20 GHz, 18.5 ps) and a fast oscilloscope (2.5 GHz, 10 GS/s) for different values of *Q* and *P*. As evident from Fig. 4(a), the Airy beam pulses have RR = 78 MHz, 2.5 GHz, 2.8 GHz and 2.5 GHz for (*Q, P*) values of (1,1), (2,16), (3,12) and (4,8), respectively. As expected, the Airy beam pulses have equal intensity for the OPO cavity synchronized (*Q* = 1, *P* = 1) to the pump RR, and a gradual decrease in pulse intensity in the train for higher values of *Q* and *P* due to the losses of intra-cavity pulses in each round trip before being amplified by the pump pulse. However, it is interesting to note that for a fixed RR (see Fig. 4(a) with RR~2.5 GHz), the increase in *Q* (here from 2 to 4) improves the relative intensity of pulses in the train, a signature of the increase in overall gain of the system.

To verify the increase in overall gain of the system we studied the power scaling characteristics of the Airy beam source configured in two different values, *Q* = 2 and *Q* = 4 to produce an output pulse train at a RR of 2.5 GHz corresponding to the *N* = 32^th^ harmonic of the pump RR with the results shown in Fig. 4(b). While operating the Airy beam OPO at *Q* = 2, we observe [see first plot of Fig. 4(b)] that the output power of the signal (Airy beam) and idler (Gaussian) radiation increase with pump power with slope efficiencies of η_Airy_ = 8.5% and η_Gauss_ = 11.7%, respectively. The source generates a maximum total output power of 0.796 W for the pump power of 4.7 W corresponding to an extraction efficiency of 16.9%. However, for the Airy beam OPO at *Q* = 4 [see second plot of Fig. 4(b)], we observe substantial enhancement in the slope efficiency of the signal (Airy beam) power, *η*_Airy_ = 16.2% without any change in the slope efficiency of the idler (Gaussian), *η*_Gauss_ = 11.6% resulting in a maximum total output power of as much as 1.194 W at an extraction efficiency of 25.4%. Such an increase in overall OPO extraction efficiency indicates the increase of overall gain of the system. Better mode matching among the interacting waves in the nonlinear crystal due to the reduced overall cavity length for *Q* = 4 as compared to *Q* = 2 can be attributed to the increase in the parametric gain and Airy beam power. This study indicates that simply by switching the cavity from *Q* = 2 to *Q* = 4, we realized high power Airy beam source in a compact design. The low operational threshold (<1 W) of the Airy beam OPO is attributed to the high parametric gain due to long crystal length (50 mm) and high intensity of the femtosecond pump laser.

Further, we studied the variation of Airy beam power with the RR for two different *Q* values (*Q* = 2 and *Q* = 3) with the results shown in Fig. 4(c). Due to the unavailability of a fast oscilloscope and a fast photo-detector, we configured the Airy beam OPO in standing wave X-cavity (not shown in the report) for *Q* = 2 (black dots) and *Q* = 3 (red dots) and extended one of the cavity mirrors (here M4) for high harmonic generation. In a ring cavity, it is more difficult to measure the increase of cavity length with high accuracy, which is the sole reason for configuring the experiment with a linear X-cavity. The RR of the Airy beam pulses is estimated simply by recording the increment in the micrometre reading attached to mirror M4 from the reading corresponding to mirror position for RR = 2.5 GHz. As evident from Fig. 4(c), the Airy beam power for *Q* = 2 (*Q* = 3) decreases from 0.99 W (1.04 W) at RR of 156 MHz (234 MHz) to 0.65 W (0.52 W) at RR = 10.3 GHz. Such decrease in output power can be attributed to the increase in round trip loss for the signal with the increase of RR. Further increase in RR up to ~100.1 GHz corresponding to *N* = 1283^rd^ harmonic of the pump RR, the Airy beam power remains almost constant for both *Q* = 3 and *Q* = 2, however, for *Q* = 3, the Airy beam power is higher by 100 mW. With increase in RR, the intra-cavity signal pulses start overlapping with each other resulting in seeding of the signal in the OPO and increase in system gain. If such gain compensates the round trip losses, then one can expect constant output power with the increase of RR. However, complete understanding of such effect required theoretical study which is beyond the scope of current report. Given that the maximum RR of the Airy beam source is determined by the temporal overlapping of the resonating pulses inside the cavity, for the Airy beam pulses having temporal width of ~639 fs, the RR of the Airy beam source can be further increased to an estimated value of ~195 GHz corresponding to the 2503^th^ harmonic of the pump RR for *Q* = 4. We also measured the power scaling property of the Airy beam source at different RR with the results shown in Fig 4(d). As expected, the slope efficiency of the Airy beam source decreases with the increase of RR. As evident from Fig. 4(d), the Airy beam source has slope efficiencies of 24%, 18.5% and 16.8% for the RR of 0.23, 5 and 100.1 GHz, respectively. Figure 4(e) shows the passive power stability (for *P* = 8 and *Q* = 4) of the Airy (red line) and Gaussian (brown line) beam measured using a power meter. As evident from Fig. 4(e), without use of any active stabilization and thermal isolation of the source from laboratory environment, the Airy beam OPO shows a power stability of 0.87% rms and 0.67% rms for Airy beam and Gaussian beam, respectively, over 30 minutes.

## Conclusions

In conclusion, we have developed femtosecond Airy beam OPO pumped with a Yb-Fiber laser at 78 MHz producing transformed limited output pulses at multi-GHz repetition rate. Based on extended phase-matching of a 50-mm long Mg:PPLN crystal configured in a novel high-harmonic and fractional cavity configuration, the Airy beam source generates 684 mW of Airy beam radiation of RR~2.5 GHz at 1525 nm and 510 mW of broadband idler radiation centred at 3548 nm in a Gaussian beam with a total extraction efficiency as high as 25.4%. The generated signal and the idler beams having BW of 3.94 nm and 94 nm, respectively. For confirmation, we have studied the peculiar properties of the Airy beam in terms of self-acceleration, non-diffraction and self-healing. The source can provide output pulses at a rep. rate as high as ~195 GHz. In this generic experimental scheme, the flexibility in the selection of *Q* (harmonic order) value gives an additional freedom to design the cavity in an optimum length to incorporate intra-cavity optical elements to produce high RR output pulses for any pump RR, time scale and wavelength.

## Additional Information

**How to cite this article:** Aadhi, A. *et al*. Multi-gigahertz, femtosecond Airy beam optical parametric oscillator pumped at 78 MHz. *Sci. Rep.* **7**, 43913; doi: 10.1038/srep43913 (2017).

**Publisher's note:** Springer Nature remains neutral with regard to jurisdictional claims in published maps and institutional affiliations.
